# How To Be a Leader: A Course for Residents

**DOI:** 10.7759/cureus.3067

**Published:** 2018-07-30

**Authors:** David A Hill, Jean-Carlos Jimenez, Stephen M Cohn, Mitchell R Price

**Affiliations:** 1 Plastic and Reconstructive Surgery, Houston Methodist Hospital, Houston, USA; 2 Surgery, Northwell Health at Staten Island University Hospital, Staten Island, USA; 3 Surgery, Staten Island University Hospital, Queens Village, USA; 4 Pediatric Surgery, Northwell Health at Staten Island University Hospital, Staten Island, USA

**Keywords:** leadership, leadership skills, leadership characteristics, residency education

## Abstract

Background: Physicians are required to assume a leadership role as part of their career. For most, this is not an innate characteristic and must be developed throughout their medical training. There are few residency courses designed to assist in the enhancement of these leadership skills. We created and implemented a novel course on leadership, utilizing weekly presentations designed to stimulate discussions and improve the leadership qualities of trainees.

Methods: Senior residents provided leadership lectures stimulated by assigned readings from the book "The Founding Fathers on Leadership." The traits and characteristics demonstrated throughout course readings and discussions were subsequently incorporated into everyday resident activities. Baseline and post-course survey responses were evaluated to assess changes in leadership qualities.

Results: Seven senior (postgraduate year (PGY) 3-5) participated as course leaders. All seven filled out pre- and post-course surveys. Seventeen junior residents (PGY 1-2) were involved as audience members. Significant pre- and post-course differences were noted in the following areas: feelings of increased encouragement of personal development (4.86 vs. 5.43, p=0.03); increased team participation in decision-making (4.00 vs. 4.57, p=0.03); increased ease of obtaining answers to difficult questions (4.57 vs. 5.23, p=0.047); increased team member work (4.86 vs. 5.71, p=0.047), and a sense of leading a more balanced life (3.86 vs. 4.43, p=0.03).

Conclusion: The initiation of a novel leadership course for senior surgical residents led to an enjoyable experience, resulting in enhanced leadership skills for all participants. We believe this process resulted in a more cohesive, efficient, communicative, and supportive residency program.

## Introduction

Physicians assume many roles throughout their careers, from educator to orator to mentor and, certainly, leaders. Doctors are often required to lead their team to deliver safe and effective medical services. Therefore, it becomes essential for these healthcare providers to develop their leadership skills so they may inspire others to deliver optimal patient care [1]. We set out to improve our trainee’s leadership skills via the implementation of a novel curriculum. We hypothesized that implementing an interactive course for trainees would result in an appreciable improvement in leadership capabilities.

## Materials and methods

In May 2016, we developed and implemented a course on leadership involving our trainees at Staten Island University Hospital, a part of Hofstra Northwell School of Medicine. The curriculum was based on readings from the book "The Founding Fathers on Leadership" by Donald Philips [2]. Senior surgical residents were randomly assigned to lead chapter discussions. Emphasis was placed on the characteristics of famous individuals instrumental in the emergence of our nation, with concrete examples of how these people demonstrated their roles as admirable leaders. Residents were also instructed to relate these skills back to how they could be incorporated into their everyday role as a physician. At the end of each presentation, the audience expressed their interpretations of the chapter to add to the overall educative experience. Residents were encouraged to incorporate concepts learned into daily activities and rounds.

Sessions took place over a three-week time span with five chapters reviewed weekly. Prior to the beginning of the course and again after completion, the senior resident presenters filled out a leadership survey (5-point Likert scale) to assess changes in their leadership capabilities. This survey was modeled after a similar leadership training course, which focused on utilizing alignment, communication, and integrity to improve leadership qualities. Course efficacy was assessed using a validated 34-question survey designed to examine participants' thoughts on certain aspects required to be a valued leader [1]. The qualities tested by this questionnaire directly correlated with the many concrete examples demonstrated by the great founding fathers of our country, as portrayed in our course textbook.

Statistical tests were two-sided and conducted at the 0.05 level of significance. Data analyses were conducted using SAS System Version 9.3 (SAS Institute Inc., Cary, NC) [3].

This study was previously presented as a three-minute "quick shot" podium presentation (Podium presentation: Hill DA, Jimenez JC, Cohn SM, Price MR. How To Be a Leader: A Course for Residents. American College of Surgeons Clinical Congress Scientific Forum; October 2017).

## Results

All seven senior residents (postgraduate year 3, 4, and 5) partook in leading course discussions. All seven course leaders completed pre and post-course surveys (100% of senior residents). A total of 17 junior residents (postgraduate year 1 and 2) were involved only as audience members. Therefore, 29% of the participants in the overall residency program acted as course leaders and completed surveys.

**Figure 1:**
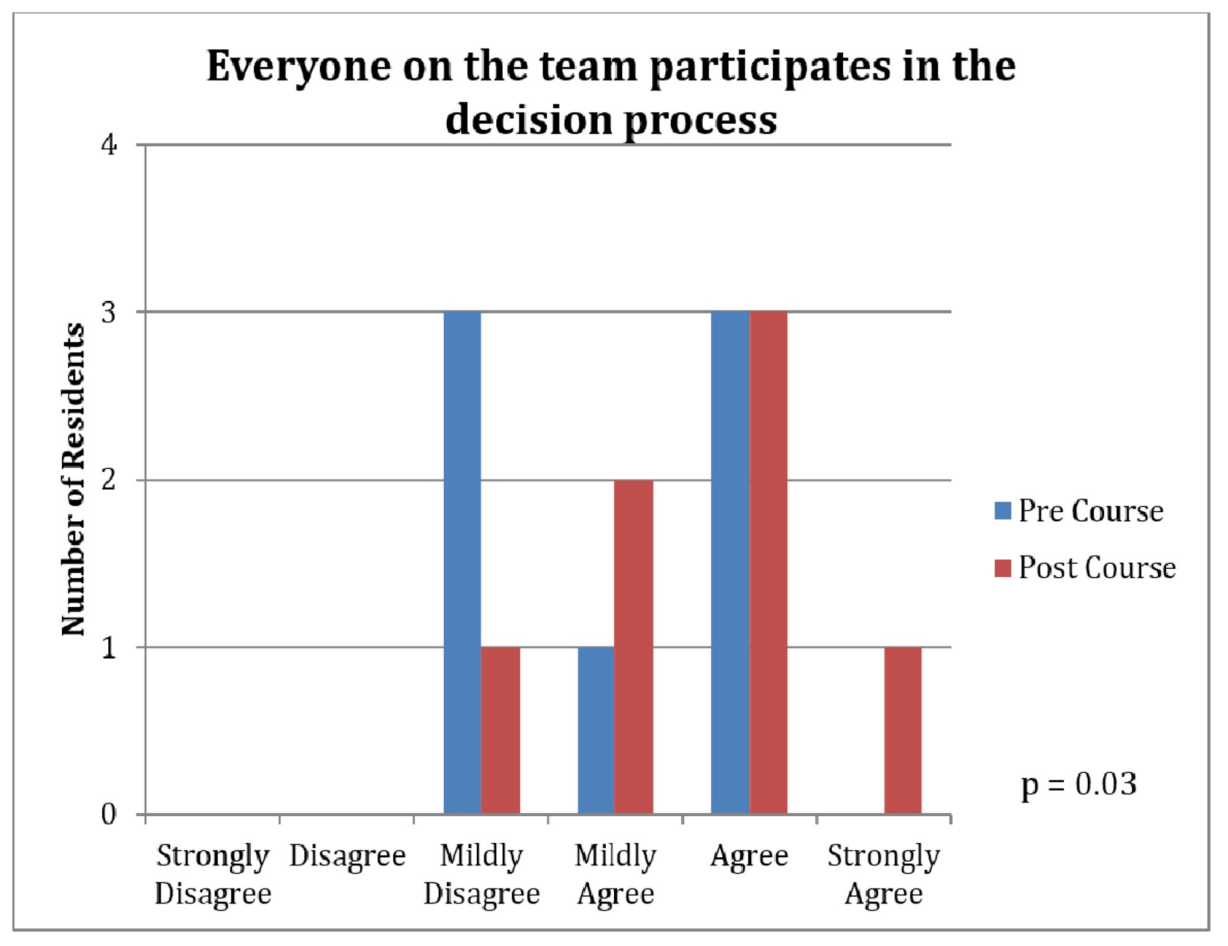
Survey Results: Inclusion of Team Members in Decision-Making

**Figure 2:**
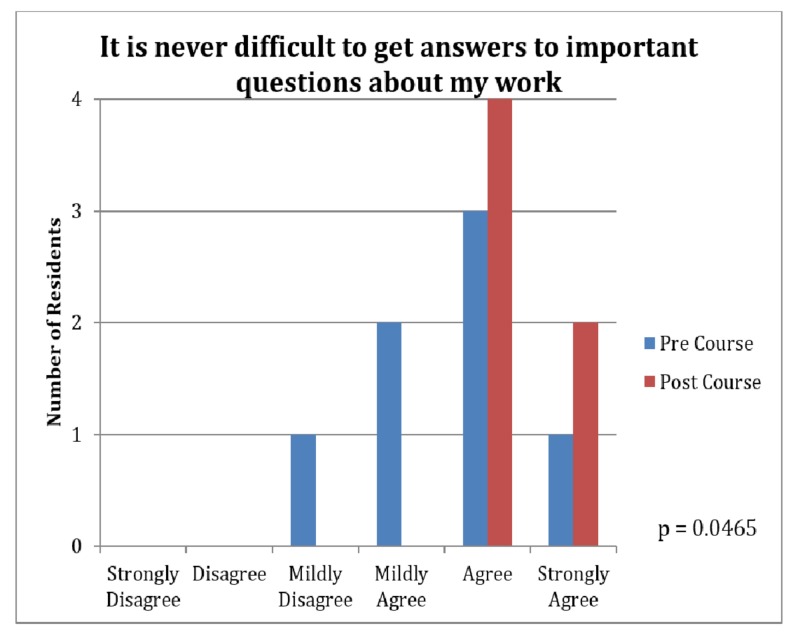
Survey Results: Difficulty of Getting Answers to Important Questions

**Figure 3:**
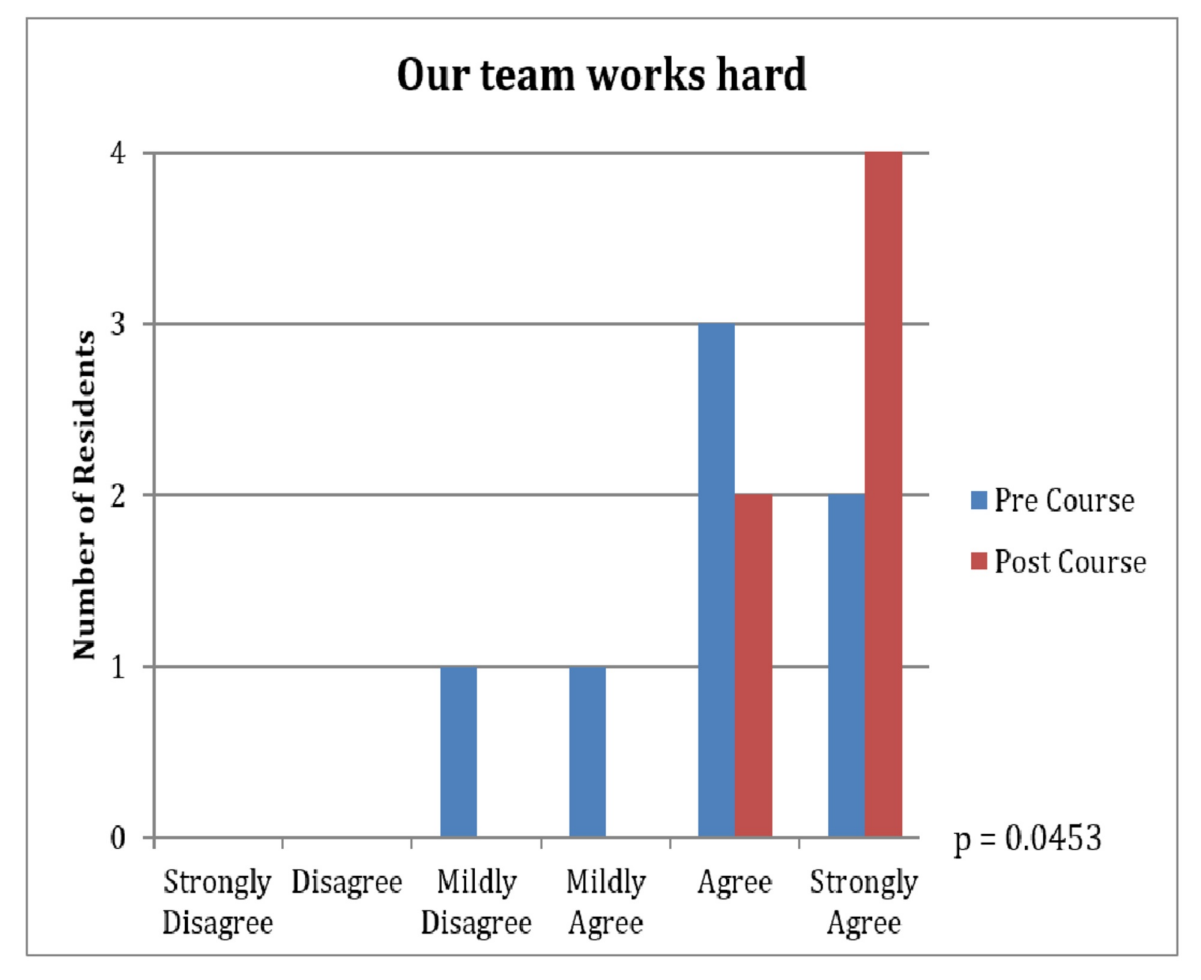
Surgery Results: The Team Works Hard

Several areas were demonstrated to have statistically significant differences between pre and post-course surveys (Table 1). During the three-week course, we appreciated an increase in team participation in decision-making (4.00 vs. 4.57, p=0.03, Figure 1) and an increase in the ability to obtain answers to difficult questions (4.57 vs. 5.29, p=0.047, Figure 2). A better acknowledgment of the teams' hard work and effort was also demonstrated (4.86 vs. 5.71, p=0.045, Figure 3).

**Table 1:** Survey Results

Question	Mean		Difference	P Value
	Pre-Course	Post-Course		
Our team members are skilled and competent	4.5714	4.8571	0.2857	0.4198
This team suffers from lack of training experience	3.00	2.5714	0.4286	0.4072
Team members strive to develop skills that can benefit the team	4.7143	4.8571	0.1429	0.3559
There are team members who have the skill or knowledge to back me up if necessary	5.2857	5.1429	0.1429	0.5628
Team members have been carefully selected to create the right mix of skills	3.5714	3.4286	0.1429	0.8688
I have challenging goals for my performance on this team	4.7143	4.5714	0.1429	0.6036
I always know what I'm supposed to be doing on this team	5.1429	5.5714	0.4286	0.0781
I know what I want to achieve on this team	5.2857	5.4286	0.1429	0.6036
Our team works hard	4.8571	5.7143	0.8571	0.0453
We are committed to superior team performance	5.4289	5.0000	0.4286	0.0781
We all accept personal responsibility for the success of the team	4.5714	4.8571	0.2857	0.4571
Team members offer help when I need it	5.4286	5.1429	0.2857	0.4973
Team members recognize that I have a life outside of work	4.0000	4.4286	0.4286	0.4072
This team often laughs together and knows how to have fun	4.8571	5.4286	0.5714	0.1030
I have at least one good friend on the team	5.4286	5.7143	0.2857	0.1723
Voicing disagreement on this team is risky	2.4286	2.5714	0.1429	0.8049
When we disagree, we usually work out our differences in an honest, healthy way	4.4286	5.2857	0.8571	0.1428
My opinions seem to count to the team	5.1429	5.1429	0.0000	1.0000
Team members cooperate with each other rather than compete	5.1429	5.2857	0.1429	0.5628
This last year, I have had opportunities to learn and grow as a member of the team	5.4286	5.8571	0.4286	0.0781
We often stop to consider how we can work better as a team	4.1429	4.2857	0.1429	0.8358
We always follow through on our plans for improving the team	4.4286	4.2857	0.1429	0.7358
I receive appropriate rewards for performing well on this team	3.5684	4.0000	0.4316	0.4674
It is never difficult to get answers to important questions about my work	4.5714	5.2857	0.7143	0.0465
I know what my team members expect of me	5.1429	5.4286	0.2857	0.1723
I'm proud to be a part of this team	5.2857	5.5714	0.2857	0.3557
My team members encourage my development	4.8571	5.4286	0.5714	0.0300
Gossip is not a problem on our team	4.4286	4.4286	0.0000	1.0000
I feel connected to my team members	4.8571	5.1429	0.2857	0.3559
I feel I can communicate to other team members	5.2857	5.2857	0.0000	1.0000
The mission and purpose of the organization makes me feel my job on the team is important	5.1429	4.8571	0.2857	0.3559
We do not have a difficult time reaching decisions	5.4286	4.7143	0.7143	0.2148
We follow a well-defined and systematic decision-making process	4.5714	4.5714	0.0000	1.0000
My team members are committed to doing quality work	4.2857	4.7143	0.4286	0.0781
Everyone on the team participates in the decision process	4.0000	4.5714	0.5714	0.0300
The team leader is skilled and experienced	5.5714	5.4286	0.1429	0.6036
The team leader gives members the freedom to make their own decision	5.1429	5.0000	0.1429	0.6036
The team leader praises or rewards members when they perform well	5.0000	5.0000	0.0000	1.0000
I always know how well I am performing on this team	5.1429	4.8571	0.2857	0.4571
The team leader gives members valuable feedback to help them improve	5.0000	5.1429	0.1429	0.3559
I have an opportunity to do my best every day on the team	5.1429	4.8571	0.2857	0.1723
We have a clear overall team purpose	5.2857	5.2857	0.0000	1.0000
Our organization fully supports this team and its mission	5.0000	4.8571	0.1429	0.6036
The team considers the impact of what we are doing on other parts of the organization	5.0000	4.7143	0.2857	0.4571
I have the materials and the equipment I need to be a strong member of the team	5.2857	5.2857	0.0000	1.0000
I am not burdened by other responsibilities that reduce my ability to contribute to the team	5.0000	4.8571	0.1429	0.7358
We have the technological resources necessary to do our work	5.1429	4.2857	0.8571	0.0781
We usually have access to the information we need	5.1429	4.4286	0.7143	0.0941
We are meeting our team objectives	5.0000	5.0000	0.0000	1.0000
So far, our team has been a great success	5.0000	5.0000	0.0000	1.0000
My team members seem to care about me as a person	4.8571	5.1429	0.2857	0.4680
I'm valued for my contribution to this team	5.0000	4.8571	0.1429	0.6891
Sign-outs can effectively convey severity of illness	4.5714	4.2857	0.2857	0.4571
Sign-outs compromise patient care	3.4286	2.8571	0.5714	0.4454
I am doing less scutt work	4.7143	5.0000	0.2857	0.3559
There is a high service to education ratio	4.5714	4.0000	0.5714	0.2308
I feel that I am taking good care of my patients	5.2857	5.2857	0.0000	1.0000
I am missing out on OR time	3.4286	2.5714	0.8571	0.3250
Overall, I am working less	2.2857	2.4286	0.1429	0.6036
I lead a more balanced life	3.8571	4.4286	0.5714	0.0300
I enjoy my work	5.2857	5.4286	0.1429	0.6036

**Figure 4:**
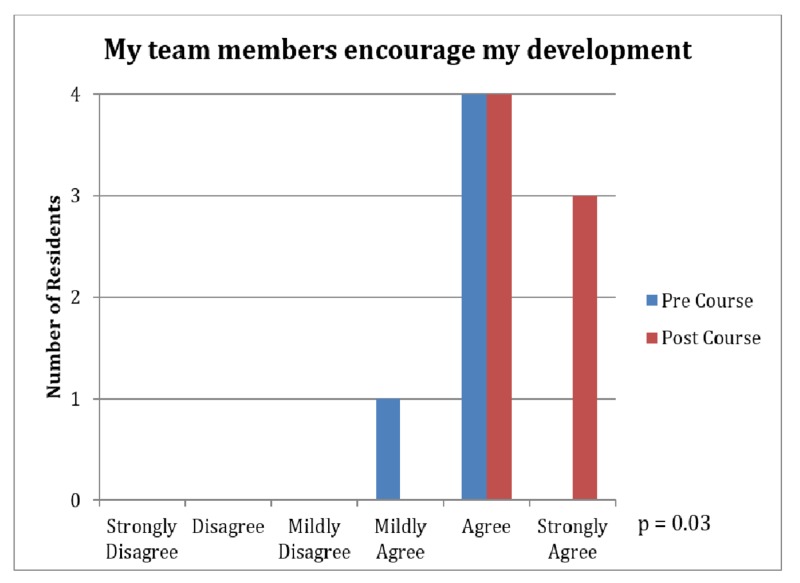
Survey Results: Encouragement of Personal Development

**Figure 5:**
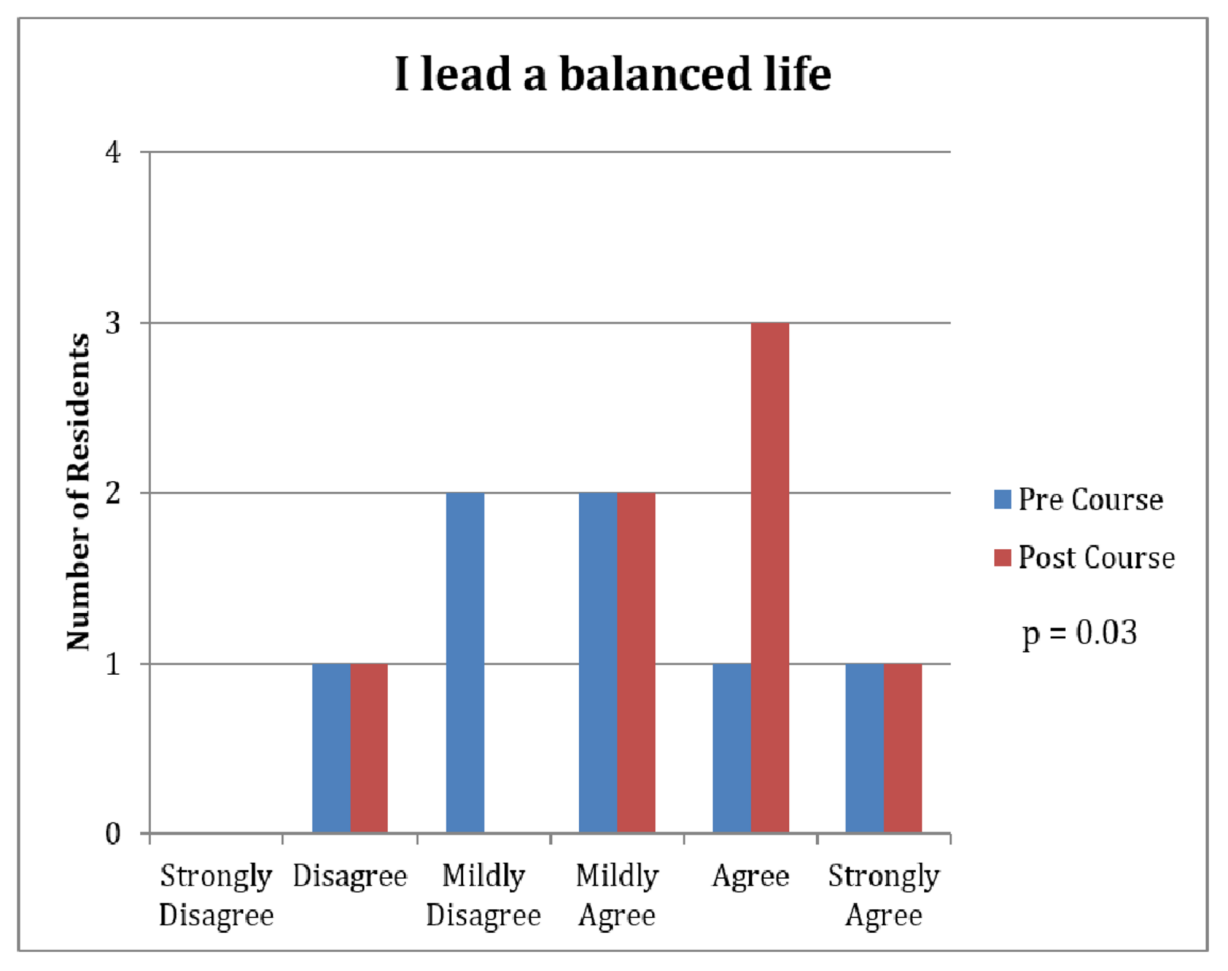
Survey Results: Leading a Balanced Lifestyle

The improved leadership qualities resulted in further lifestyle benefits as well. Team members noted an increased sense of encouragement for personal development (4.86 vs. 5.43, p=0.03, Figure 4). Another significant finding was that the course resulted in a lifestyle change that residents perceived as leading to a more balanced life (3.86 vs. 4.43, p=0.03, Figure 5).

## Discussion

For many, taking on the role of leader is an acquired trait that takes many years of practice to master. Asserting leadership is an essential skill for successful physicians working in the healthcare industry [4-5]. Throughout their training, residents are required to assume the role of leaders. However, few enter into these positions as accomplished individuals with advanced leadership skills. Put another way, while there may occasionally be a “born leader” (e.g. Abraham Lincoln), it is a learned behavior for the rest of us [5-7]. We believe that the acquisition of strong leadership skills should be emphasized during the teaching of medical professionals.

The implementation of a course for postgraduate trainees focusing on leadership resulted in an entertaining and educational experience. The weekly readings from the book "The Founding Fathers on Leadership" by Donald Philips highlighted specific characteristics required to be a successful leader [2]. These examples led to discussions concerning personal experiences in which these skills were essential in providing patient care. They also led to significant daily changes in our residency program. One such instance was developed from chapter six, entitled "Travel With the Troops." In this section, it describes how George Washington would set up daily meetings with his military leaders to first open up direct lines of communication so they could easily and openly relay one another's concerns. However, this also allowed Washington to better understand his leaders' ideas and ways of thinking and to truly understand and get to know the people he was commanding. Conversely, it also gave his subordinates a sense that their ideas were important and that they were being included in overall decision-making. To emulate this in our residency program, we initiated a daily morning conference for which all new surgical admissions and consults are to be discussed with the entire residency program and attending staff. This allowed for open discussions with the goal of providing optimal patient care. It also had the secondary effects of garnishing feelings of inclusion, as well as a very educational experience, in that it allowed for discussions and, sometimes, debates concerning various ways of approaching certain clinical scenarios.

A key component of the course was the comradery amongst the people involved. Our residency program is fairly small and thus each session consisted of only 20 to 30 people. All participants were already very familiar with one another, and this fostered an open learning environment. The willingness to share personal examples of leadership techniques proved to be beneficial for the education of the entire group.

A significant improvement was noted in our survey regarding inclusiveness in decision-making. This was specifically evident in changes inspired by the course in our daily rounds. We slowly transitioned from a single speaker (the chief resident) to more of a brainstorming type of atmosphere, where all residents were allowed to voice their opinions and concerns regarding patient plans. This increased inclusion and further fueled residents’ desire to work hard and continue to provide the high-quality care our patients expect. In the end, through open discussions concerning leadership and the practicing of these qualities during daily rounds, the residency program developed a tighter bond and further advanced important leadership characteristics necessary to become successful physicians.

Although the small residency program size may have assisted in decreasing the anxiety regarding sharing personal details, this small sample size was also one of the biggest limitations to the study. Being limited to the number of residents in our program led to only having seven before and after surveys to compare. Thus, in order to increase the study power, it will be necessary to expand to include other residency programs in our institution or attempt a multicenter course.

## Conclusions

The initiation of a novel leadership course for senior surgical residents led to an enjoyable experience, resulting in enhanced leadership skills for all participants. We believe that this process resulted in a more cohesive, efficient, communicative, and supportive residency program.
